# Quiet Quitting and Counterproductive Work Behavior: Evidence from a Cross-Sectional Study of Healthcare Professionals Across Organizational and Interpersonal Outcomes

**DOI:** 10.3390/healthcare14172726

**Published:** 2026-08-26

**Authors:** Danijela Ilic, Violeta Domanovic, Marko Slavkovic

**Affiliations:** 1 Health Center Rekovac, Dr Johana Johanesona bb, 35260 Rekovac, Serbia; dacapedijatrija6@gmail.com; 2 Department of Management and Business Administration, Faculty of Economics, University of Kragujevac, Liceja Knezevine Srbije 3, 34000 Kragujevac, Serbia; vterzic@kg.ac.rs

**Keywords:** quiet quitting, counterproductive work behavior, healthcare workers, work-related outcomes, social exchange theory

## Abstract

**Background/Objectives**: Quiet quitting has become an increasingly prominent workplace phenomenon, particularly within healthcare organizations. Although previous research has primarily focused on the factors associated with its emergence, considerably less attention has been devoted to its relationships with undesirable workplace behaviors. This study examined the relationship between quiet quitting and selected forms of counterproductive work behavior among healthcare professionals. **Methods**: A cross-sectional study was conducted among healthcare professionals employed in public healthcare institutions in Central Serbia. Data were collected between February and March 2026 using a structured questionnaire. Quiet quitting was assessed using the Quiet Quitting Scale (QQS), while counterproductive work behavior was measured through selected dimensions of the Counterproductive Work Behavior Checklist (CWB-C). The data were analyzed using partial least squares structural equation modeling (PLS-SEM). **Results**: Quiet quitting was positively and statistically significantly associated with counterproductive work behavior directed toward the organization, counterproductive work behavior directed toward coworkers, and withdrawal behavior. The strongest relationship was observed for counterproductive work behavior directed toward coworkers. No statistically significant relationship was identified between quiet quitting and sabotage. Multigroup analysis indicated differences in several relationships across gender and age groups. **Conclusions**: The findings indicate that quiet quitting is associated with several forms of counterproductive work behavior in healthcare organizations. The differentiated pattern across organizational, interpersonal, withdrawal-related, and sabotage outcomes supports the view that quiet quitting and counterproductive work behavior are conceptually related but not equivalent.

## 1. Introduction

Healthcare organizations depend strongly on the attitudes and behavior of their employees. Recent evidence has drawn particular attention to quiet quitting among nurses and other healthcare workers [1]. More broadly, employee attitudes such as job satisfaction are relevant to service-related outcomes [2]. Quiet quitting has been discussed as a particularly important issue in healthcare [3], while recent healthcare research has continued to clarify its meaning and relevance in hospital settings [4]. Counterproductive work behavior represents another important workplace phenomenon and may vary according to individual and demographic characteristics [5]. Managerial perspectives have also become part of the growing discussion on how quiet quitting is understood and addressed in organizations [6].

Social exchange theory provides a useful framework for considering these workplace responses and has been widely applied in organizational research [7]. Its central premise is that relationships are maintained through reciprocal exchanges in which individuals evaluate the benefits and costs associated with their interactions [8]. In organizational settings, employees may respond to support, fair treatment, recognition, and fulfilled expectations with positive attitudes and behaviors, whereas unfavorable exchanges may be associated with negative responses [9]. These considerations are also relevant in healthcare organizations, where responsible management of employees represents an important aspect of sustainable organizational functioning [10].

Quiet quitting has received increasing attention among healthcare professionals, particularly nurses, whose work environment may contribute to reduced engagement [11]. Studies have also linked quiet quitting among nurses with turnover intention [12], while the development of the Quiet Quitting Scale provided a specific instrument for assessing phenomenon [13]. In healthcare settings, quiet quitting has been examined in relation to quality of work life [14], workplace bullying [15], and moral resilience [16]. Evidence from Serbia has also shown growing interest in the phenomenon among employees [17]. Importantly, quiet quitting does not necessarily involve an intention to harm the organization; rather, it mainly reflects reduced motivation, initiative, and discretionary contribution while employees remain within their formal employment role [18].

Quiet quitting has also been considered in relation to a broader set of employee and organizational conditions. Burnout has been discussed as one of its psychological drivers [19], while research in hospital settings has examined the roles of conflict, organizational support, and professional engagement [20]. Other studies have considered the causes and possible managerial responses to quiet quitting [21], as well as its relationships with employee performance and well-being [22]. In healthcare, innovation support has been associated with lower quiet quitting and more favorable innovative behavior [23], while other evidence has emphasized possible implications for healthcare organizations and services [24]. Organizational culture and management practice have also been examined in this context [25]. A recent systematic review confirms the growing research interest in the health sector [26].

Counterproductive work behavior refers to voluntary employee actions that violate organizational norms and may harm the organization or individuals working within it [27]. Recent reviews show that CWB is a multidimensional workplace phenomenon associated with a broad range of individual and organizational factors [28]. Work stress, organizational justice, and demographic characteristics have also been considered in relation to CWB [29]. Person-organization misfit represents another possible context for counterproductive responses [30], while organizational justice and job satisfaction have been linked to CWB in previous research [31]. Workplace deviance may be distinguished according to its target and behavioral form [32], including the classical distinction between organizational and interpersonal deviance [33]. Deviant behavior has also been interpreted as a possible form of organizational resistance [34]. In healthcare, undesirable and disruptive workplace behaviors have been documented as relevant organizational and interpersonal problems [35,36], and perceived injustice has been associated with CWB among healthcare workers [37]. More broadly, workplace deviance research indicates that such behaviors may have specific organizational antecedents and outcomes [38], while interpersonal conflict is particularly relevant to person-directed CWB [39]. In its more pronounced forms, and in line with the assumptions of social exchange theory, situations in which employees perceive costs to outweigh benefits may result in the emergence of substantially more aggressive forms of counterproductive work behavior [19].

Quiet quitting has sometimes been positioned within the broader domain of counterproductive work behavior [40]. However, this conceptual proximity does not imply that quiet quitting and CWB are equivalent constructs. Quiet quitting primarily reflects the restriction of motivation, initiative, and discretionary contribution while employees remain within their formal job requirements, whereas CWB represents a broader domain of voluntary undesirable behaviors directed toward the organization or its members. The CWB-C framework further distinguishes different behavioral targets and domains, including Withdrawal and Sabotage [41]. The distinction between quiet quitting and Withdrawal requires particular attention because both involve some form of disengagement. Quiet quitting mainly concerns withholding discretionary contribution, whereas Withdrawal captures behaviors that reduce actual work attendance or working time, such as unauthorized lateness, unjustified absence, longer-than-permitted breaks, and leaving work earlier than allowed [41]. The constructs may therefore be conceptually related but not equivalent.

From the perspective of social exchange theory, quiet quitting and CWB can be understood as conceptually related but behaviorally different responses associated with the quality of the employee-organization exchange. When employees perceive the exchange relationship as unfavorable, they may reduce their willingness to contribute beyond formal job requirements [31]. Quiet quitting reflects such a restriction of discretionary contribution, whereas CWB includes a broader range of behaviors directed toward the organization or other organizational members [41]. The principle of reciprocity therefore provides a theoretical basis for expecting associations between quiet quitting and selected forms of CWB without implying that these behaviors are identical or that one necessarily develops into another.

The healthcare context is particularly relevant for examining these relationships because healthcare work depends strongly on coordination and cooperation among professionals. When an employee reduces initiative, avoids additional responsibilities, or limits contribution to the formally required minimum, some work demands may be transferred to coworkers. This may increase interpersonal strain and provide a contextual basis for expecting a relationship between quiet quitting and counterproductive behavior directed toward other employees [35,36]. This mechanism is also consistent with evidence linking interpersonal conflict with counterproductive behavior [39]. At the organizational level, restriction of discretionary contribution may coexist with behaviors that negatively affect organizational functioning. Because negative reciprocity may also be expressed through more active organization-directed behavior, the study additionally examines whether the positive association extends to the more distal domain of Sabotage [31,41].

Although scholarly interest in quiet quitting has increased substantially, the healthcare literature remains strongly focused on its antecedents and related working conditions [26]. Less attention has been devoted to its relationship with other undesirable workplace behaviors. Moreover, even when quiet quitting is positioned within the broader CWB domain [40], this does not establish whether it is similarly related to organizational, interpersonal, withdrawal-related, and more intentionally CWB. Accordingly, the aim of this study is to examine whether, and to what extent, quiet quitting is associated with selected forms of counterproductive work behavior among healthcare professionals.

**Figure 1:**
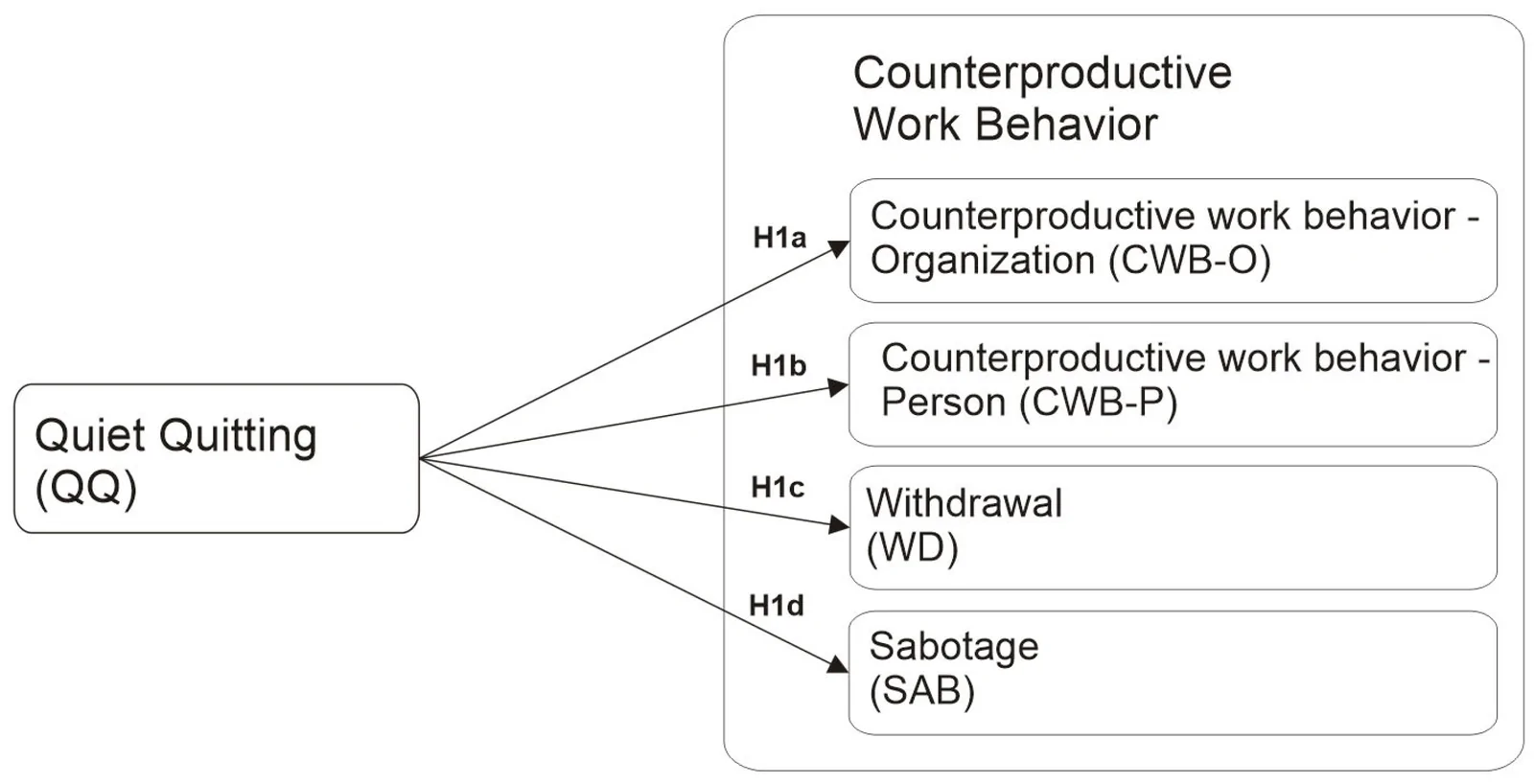
Conceptual research model.

In accordance with the aforementioned, the subsequent hypotheses and research framework were formulated (Figure 1).

**H1a:**  *Quiet quitting is positively associated with counterproductive work behavior directed toward the organization.*

**H1b:**  *Quiet quitting is positively associated with counterproductive work behavior directed toward other employees.*

**H1c:**  *Quiet quitting is positively associated with Withdrawal.*

**H1d:**  *Quiet quitting is positively associated with Sabotage.*

## 2. Materials and Methods

### 2.1. Participants and Procedures

An empirical study was conducted using a cross-sectional research design. The target population comprised healthcare professionals employed in public healthcare institutions in Central Serbia. A convenience sampling approach was used. Potential respondents were approached in ten public healthcare institutions, including institutions providing primary, secondary, and tertiary healthcare. Recruitment was conducted on different weekdays in order to broaden the coverage of healthcare professionals available during the data collection period. To reduce the potential for selection bias and increase sample heterogeneity, respondents were recruited across multiple public healthcare institutions and on different weekdays.

Data collection was carried out from late February to March 2026 using printed questionnaire. Prior to approaching potential respondents, verbal permission was obtained from the management of the participating healthcare organizations. The study was conducted in accordance with the ethical principles of the Declaration of Helsinki. The introductory page of the questionnaire contained an informed consent statement. Potential respondents were informed about the academic purpose of the study, the voluntary nature of participation, anonymity, and the confidentiality of the collected data. The questionnaire did not include highly sensitive personal information or information that could directly identify participants. Demographic information was used only for statistical analysis. Respondents could request additional information or clarification regarding the study or questionnaire content both before and during completion of the survey. Informed consent was obtained from all participants prior to data collection.

A minimum target of 500 respondents was established for data collection. Once this number had been exceeded, data collection was closed. Because the number of healthcare professionals who declined participation was not systematically recorded, a response rate could not be calculated. The final usable sample included in the statistical analysis comprised 517 healthcare professionals.

### 2.2. Measurements

Data were collected using a structured questionnaire consisting of three sections. The first section assessed quiet quitting using the Quiet Quitting Scale (QQS) developed by Galanis et al. [13]. The QQS includes statements related to motivation, initiative, willingness to undertake additional work responsibilities, and the extent to which employees invest effort beyond formal job requirements. The original instrument was translated into Serbian using forward- and back-translation procedure [41]. The translated questionnaire was reviewed for suitability in the Serbian context and was pretested with 30 respondents before the main data collection to assess clarity and comprehensibility and to identify whether alternative wording was required. The scale includes two response formats: agreement-based items rated on a five-point Likert scale from 1 (“strongly disagree”) to 5 (“strongly agree”), and frequency-based items rated from 1 (“never”) to 5 (“always”).

The second section of the questionnaire was intended to assess counterproductive work behavior among healthcare professionals. In this study, counterproductive work behavior was measured using the Counterproductive Work Behavior Checklist (CWB-C) developed by Spector et al. [42]. The official Serbian-language version of the instrument was used (“Copyright Paul E. Spector, All rights reserved. Translated into Serbian language in October 2018”). The study was not designed to examine the complete dimensional structure of the CWB-C but rather theoretically selected CWB outcomes considered relevant to quiet quitting in healthcare. CWB-Organization and CWB-Person were selected to capture the broader organizational and interpersonal targets of counterproductive behavior. Withdrawal was included because of its theoretical proximity to quiet quitting while representing more explicit reductions in required work participation, whereas Sabotage was included as a more severe and behaviorally distinct organizational CWB outcome [42]. Production Deviance was not included because its content concerning intentionally reduced or inadequate work performance may overlap with aspects of quiet quitting and therefore blur the distinction between predictor and outcome. Theft was excluded because of its weaker theoretical proximity to quiet quitting and the particularly sensitive legal and professional nature of self-reported theft behavior in healthcare settings.

The frequency of the selected CWB behaviors was assessed using a five-point scale ranging from 1 (“never”) to 5 (“very often”), with higher scores indicating a higher level of counterproductive work behavior. The distinction between quiet quitting and Withdrawal was also reflected in the content of the measures. The retained QQS indicators primarily captured reduced motivation and initiative, withholding of ideas, and restriction of effort to minimum job requirements. In contrast, the Withdrawal items referred to unauthorized lateness, unjustified absence, longer-than-permitted breaks, and leaving work earlier than allowed. Thus, although some behavioral proximity was expected, the measures captured different forms of reduced work involvement.

The third section collected demographic information on respondents’ gender and age. These variables were included to describe the sample and to examine possible differences in the relationships across demographic groups. The questionnaire was organized into three separate sections in order to clearly distinguish the measurement of quiet quitting, counterproductive work behavior, and demographic characteristics and to reduce potential response-related bias.

### 2.3. Data Analysis

Statistical data analysis was performed using partial least squares structural equation modeling (PLS-SEM) in SmartPLS 4 [43]. The analysis was conducted in two stages, involving the evaluation of the measurement model and the structural model. As part of the measurement model evaluation, the reliability and validity of the constructs were assessed. Reliability was examined using Cronbach’s alpha and composite reliability (CR), while convergent validity was evaluated based on average variance extracted (AVE) and indicator loadings. Following the assessment of convergent validity, discriminant validity was examined using the heterotrait–monotrait ratio (HTMT). Indicator evaluation followed the recommendations for PLS-SEM measurement assessment provided by Hair et al. [44].

The structural model was assessed based on coefficients of determination (R^2^), structural path coefficients, and their statistical significance. Bootstrapping with 5000 sub-samples and a two-tailed test was used, with statistical significance established at *p* < 0.05. Potential endogeneity was assessed using the Gaussian copula approach by including the corresponding copula term for quiet quitting as an additional control-function predictor in each structural equation [45]. An approximate joint Wald-type statistic was also considered as an additional diagnostic of the joint relevance of the copula terms.

**Figure 2:**
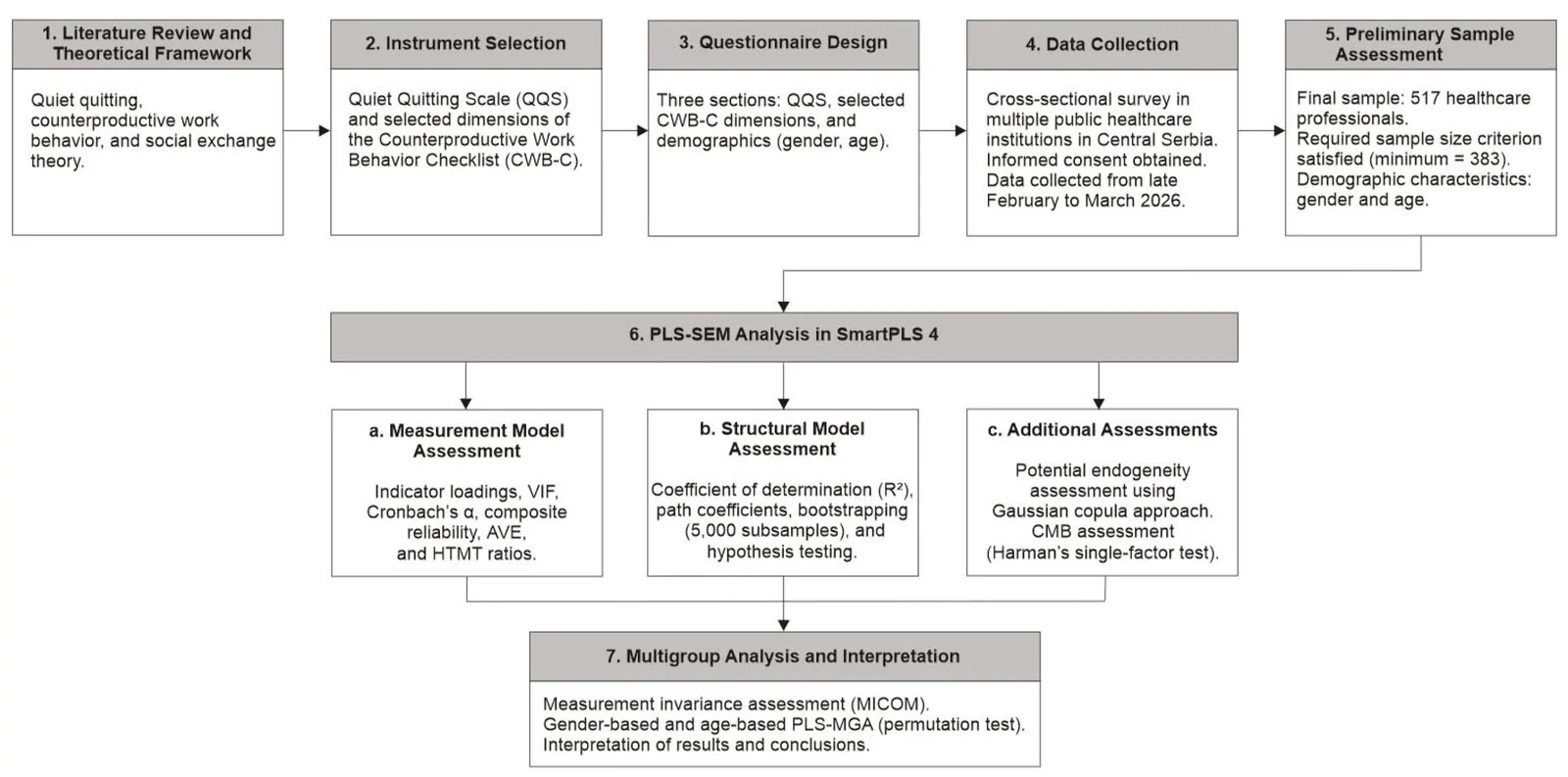
Flowchart of the study design and analytical procedure.

Because predictor and outcome variables were collected from the same respondents using a single questionnaire, potential common method bias was additionally examined using Harman’s single-factor test [46]. All questionnaire items were entered simultaneously into an unrotated principal component analysis. Prior to multigroup analysis, measurement invariance across gender and age groups was assessed using the Measurement Invariance of Composite Models (MICOM) procedure. Group differences in structural path coefficients were subsequently assessed using formal PLS-MGA/permutation testing [44]. The overall study design and analytical procedure are summarized in Figure 2.

## 3. Results

### 3.1. Respondents’ Profiles

The final sample comprised 517 healthcare professionals (Table 1). As an additional assessment of sample adequacy, the required sample size was estimated using Cochran’s formula with a 95% confidence level and a 5% margin of error. According to the Health Statistical Yearbook of the Republic of Serbia 2024, 110,193 employees worked in healthcare institutions [47]. Based on this population size, the minimum required sample was estimated at 383 respondents. The obtained sample of 517 respondents therefore exceeded this minimum. Female respondents constituted the majority of the sample (85.11%). This predominance of women is not unexpected in the healthcare context. According to the Health Statistical Yearbook of the Republic of Serbia 2024, women account for 68% of medical doctors in Serbia [47], while nursing is also strongly female-dominated internationally, with women accounting for approximately 85% of the global nursing work-force [48]. These figures are not interpreted as evidence that the present sample re-produces the occupational composition of the national healthcare workforce, but they provide context for the strong female representation in the sample. With regard to age, respondents aged 40 years or younger represented 42.7% of the sample, followed by those aged 41–50 years (29.21%) and respondents older than 51 years (28.05%).

### 3.2. Measurement Model Assessment

The measurement model was assessed in terms of reliability and validity (Table 2). Cronbach’s alpha values ranged from 0.700 to 0.916, exceeding the recommended threshold proposed by Nunnally and Bernstein [49], while composite reliability values ranged from 0.745 to 0.919 [50]. These findings indicate a satisfactory internal consistency for all constructs.

Convergent validity was assessed using average variance extracted (AVE). All AVE values exceeded the recommended threshold of 0.50 [51], ranging from 0.557 to 0.652. Indicator reliability was assessed based on outer loadings. Following Hair et al. [44], loadings of approximately 0.708 or higher are generally desirable, while indicators with somewhat lower loadings may be retained when their theoretical relevance and the reliability and convergent validity of the corresponding construct remain satisfactory. Most retained indicators exceeded the recommended value. Three retained indicators had loadings slightly below 0.708: SAB03 (0.695), CWB-O02 (0.698), and CWB-O07 (0.705). They were retained because their loadings were close to the recommended value and the respective constructs demonstrated satisfactory reliability and convergent validity. Sabotage had α = 0.700, CR = 0.745, and AVE = 0.620, while CWB-Organization had α = 0.801, CR = 0.803, and AVE = 0.557.

Indicators with substantially lower loadings were considered for removal. Within the Quiet Quitting scale, indicator QQ04 (“If a colleague can do some of my work, then I let him/her do it.”) was excluded. Two indicators were removed from Counterproductive Work Behavior–Organization construct (“Daydreamed rather than did your work” and “Failed to report a problem so it would get worse”), while “Hid something so someone at work couldn’t find it” was also excluded from the Counterproductive Work Behavior–Person construct. Their removal was based primarily on comparatively weak measurement performance. From a contextual perspective, allowing a colleague to perform part of one’s work may not necessarily indicate quiet quitting in healthcare settings where teamwork and task sharing are common. Similarly, the removed CWB items represent specific behaviors that may not reflect the broader organizational or interpersonal dimensions equally across healthcare occupations. Removal therefore does not imply that these behaviors are theoretically irrelevant. Indicator-level collinearity was assessed using outer VIF values. All reported outer VIF values were below 5, ranging from 1.306 to 3.391 [52] (Table 2).

Discriminant validity was assessed using the heterotrait–monotrait ratio (HTMT). The results are presented in Table 3. According to the recommendations of Henseler and Ringle [53], discriminant validity is considered satisfactory when HTMT values are below the threshold of 0.85. The findings indicate that all HTMT coefficients were below the recommended limit. Of particular relevance to the conceptual distinction between Quiet Quitting and Withdrawal, the HTMT value between these constructs was 0.754. Although this value reflects their expected conceptual proximity, it remains below the adopted threshold and provides empirical support for treating the constructs as distinct in the present model.

Based on these results, the measurement model demonstrated satisfactory discriminant validity. Potential common method bias was additionally assessed using Harman’s single-factor test [46]. The analysis included all 28 questionnaire items and all 517 respondents. The first unrotated component explained 39.81% of the total variance, while five components had eigenvalues greater than 1. No single factor therefore accounted for the majority of the variance, suggesting that common method bias was not a major concern according to this diagnostic test.

### 3.3. Structural Model Assessment

The coefficients of determination (R^2^) were obtained from the Gaussian-copula-corrected structural model. The model explained 48.4% of the variance in Counterproductive Work Behavior–Person, 43.8% of the variance in Withdrawal, 36.9% of the variance in Counterproductive Work Behavior–Organization, and 17.3% of the variance in Sabotage (Table 4). The highest proportion of explained variance was therefore observed for counterproductive work behavior directed toward other employees.

Before testing the structural relationships, potential endogeneity was assessed using the Gaussian copula approach [45]. The corresponding copula term for quiet quitting was included as an additional control-function predictor in each structural equation. The copula terms were statistically significant for the CWB-O (β = −0.871, *p* = 0.044) and CWB-P (β = −1.075, *p* = 0.016), marginally significant for Withdrawal (β = −0.808, *p* = 0.056), and not statistically significant for Sabotage (β = −0.165, *p* = 0.749) (Table 5). The approximate joint Wald-type statistic for the four copula terms was also statistically significant, W(4) = 13.659, *p* = 0.0085, indicating that potential endogeneity was relevant to at least some of the examined structural relationships. Accordingly, the interpretation of the findings is based on the model incorporating the Gaussian copula correction.

**Figure 3:**
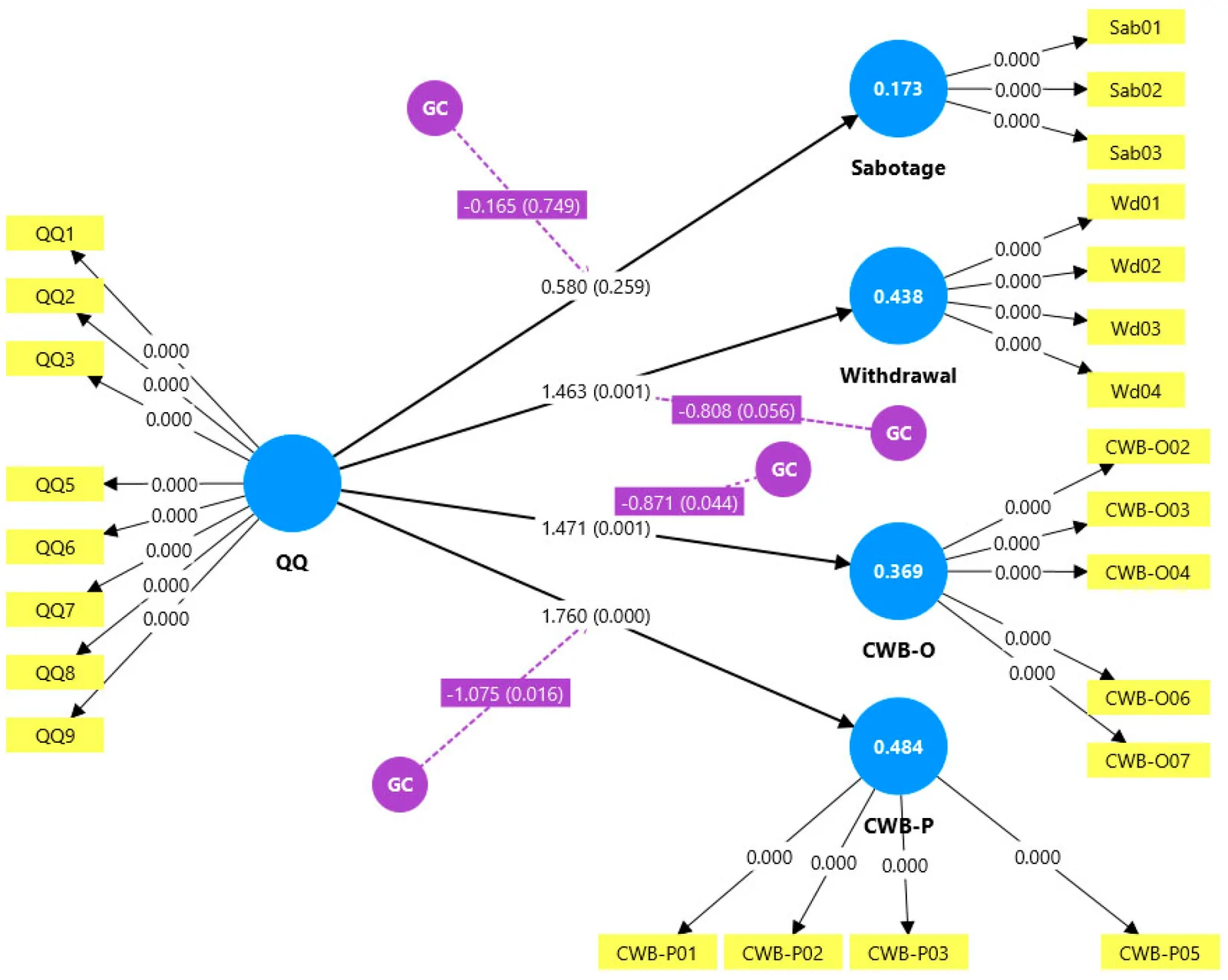
Final Gaussian-copula-corrected structural model.

The structural relationships were assessed using bootstrapping with 5000 subsamples and percentile confidence intervals. Quiet quitting was positively and significantly associated with CWB-Organization (β = 1.471, *p* < 0.001), supporting H1a. A positive and statistically significant relationship was also identified with CWB-Person (β = 1.760, *p* < 0.001), supporting H1b. This was the strongest relationship observed in the model. Quiet quitting was positively and significantly associated with Withdrawal (β = 1.463, *p* < 0.001), providing support for H1c. In contrast, the relationship with Sabotage was not statistically significant (β = 0.580, *p* = 0.259); therefore, H1d was not supported (Table 6). Because quiet quitting and its corresponding Gaussian copula term were jointly estimated in each corrected structural equation, the reported coefficients are standardized regression estimates from a multiple-predictor, endogeneity-corrected specification and should not be interpreted as zero-order correlations (Figure 3). Consequently, standardized coefficients above 1.0 do not represent calculation or reporting errors.

Prior to multigroup analysis, measurement invariance across gender and age groups was assessed using the MICOM procedure. Full measurement invariance was established for Quiet Quitting, CWB-Organization, CWB-Person, and Withdrawal across both grouping variables. Compositional invariance was not established for Sabotage in Step 2. Therefore, multigroup comparisons involving Sabotage were not interpreted.

In the next stage multigroup analysis was performed to examine whether the relationship between quiet quitting and different forms of counterproductive work behavior varies according to respondents’ gender and age. Respondents were divided into two gender groups and three age categories: under 40 years, 41–50 years, and over 51 years. The results are presented in Table 7. Formal PLS-MGA/permutation testing indicated statistically significant gender differences in the relationships QQ → CWB-O, QQ → CWB-P, and QQ → WD. The group-specific estimates were stronger among women, whereas the corresponding paths were not statistically significant among men. Age-based comparisons also indicated statistically significant differences in these three relationships. QQ → CWB-O and QQ → WD were statistically significant among respondents aged 40 years or younger, while QQ → CWB-P was statistically significant among respondents aged 40 years or younger and among those aged 41–50 years. The strongest group-specific relationship with CWB-P was observed among respondents aged 41–50 years. Multigroup results involving Sabotage are shown in Table 6 for transparency but are not interpreted because compositional invariance was not established for this construct.

## 4. Discussion

The aim of this study was to examine whether, and to what extent, quiet quitting is associated with selected forms of counterproductive work behavior among healthcare professionals. The findings showed significant positive relationships between quiet quitting and CWB directed to-ward the organization, CWB directed toward other employees, and Withdrawal. Accordingly, H1a, H1b, and H1c were supported. In contrast, the relationship between quiet quit-ting and Sabotage was not statistically significant, and H1d was not supported. This differentiated pattern indicates that quiet quitting is not uniformly associated with all forms of counterproductive work behavior. The significant copula terms for CWB-Organization and CWB-Person indicate that the endogeneity adjustment was relevant for these relationships; therefore, the following interpretation is based on the corrected specification. These findings are generally consistent with social exchange theory, according to which employees may respond to an unfavorable exchange relationship by adjusting their attitudes and behavior toward the organization and its members. However, the results also suggest that such responses may differ in their form and target. The strongest relationship was identified between quiet quitting and CWB-Person. In healthcare organizations characterized by interdependent work, high workloads, stress, and demanding working conditions, a reduced willingness to assume additional responsibilities may redistribute work demands among colleagues and increase interpersonal strain. This provides a possible contextual explanation for the strong association with CWB directed toward coworkers [35,36].

The positive relationship between quiet quitting and CWB-Organization supports H1a. This finding is consistent with previous arguments that unfavorable perceptions of the employee–organization relationship may be associated with lower organizational commitment and undesirable workplace behavior [5,31]. Employees who engage in quiet quitting remain within their formal employment role but reduce their willingness to invest discretionary effort. Such reduced engagement may coexist with behaviors that negatively affect organizational processes, work efficiency, and organizational resources.

The positive relationship between quiet quitting and Withdrawal provides support for H1c. The conceptual proximity of these constructs makes this association understandable, but they should not be treated as equivalent. Quiet quitting mainly concerns reduced motivation, initiative, and discretionary contribution while employees remain within the formal boundaries of their job requirements [13]. In contrast, the Withdrawal dimension used in this study captures behaviors that reduce required work attendance or working time, such as unauthorized lateness, absence, extended breaks, and early departure [42]. Thus, quiet quitting primarily reflects the withholding of discretionary effort, whereas Withdrawal reflects a reduction in required work participation. The HTMT value of 0.754 provides additional empirical support for treating the constructs as distinct, although their conceptual proximity is expected. The observed relationship therefore indicates that employees reporting higher levels of quiet quitting also tend to report more explicit withdrawal behaviors, without implying that quiet quitting necessarily precedes or causes Withdrawal.

H1d was not supported, as no statistically significant relationship was identified between quiet quitting and Sabotage. This finding is theoretically relevant because it indicates an important behavioral boundary of quiet quitting. Quiet quitting mainly reflects a reduction in discretionary contribution and does not necessarily involve an intention to harm the organization [18]. Sabotage, in contrast, represents a more explicit and intentionally harmful form of counterproductive behavior [42]. From the perspective of social exchange theory, employees may respond to an unfavorable exchange relationship by restricting effort or initiative without engaging in deliberately destructive behavior. An alternative explanation is that Sabotage is a more distal and severe behavioral response that may depend on additional factors, such as stronger perceptions of injustice, anger, conflict, or other individual and organizational conditions that were not examined in this study. The absence of a significant relationship therefore helps define the behavioral boundary of quiet quitting rather than merely representing an unsupported hypothesis.

The multigroup analysis revealed statistically significant gender differences in the relationships between quiet quitting and counterproductive work behavior directed toward the organization, counterproductive work behavior directed toward other employees, and Withdrawal. These relationships were stronger among women, whereas the corresponding group-specific paths were not statistically significant among men. This finding differs from previous studies reporting that men tend to exhibit higher overall levels of counter-productive work behavior [5,27]. However, the present results concern differences in the relationship between quiet quitting and CWB rather than differences in the overall level of CWB, and these findings are therefore not necessarily contradictory. Women constituted 85.11% of the sample. A strong female presence is not unexpected in the healthcare context. Nevertheless, because professional category was not collected, it is not possible to determine whether the observed gender differences partly reflect occupational composition. The gender-based findings should therefore be interpreted with caution.

Interesting differences were also observed across age groups. The relationship between quiet quitting and CWB-Organization and the relationship be-tween quiet quitting and Withdrawal were statistically significant among employees aged 40 years or younger, while the strongest relationship between quiet quitting and CWB-Person was observed among respondents aged 41–50 years. These findings are generally consistent with previous evidence indicating age-related differences in counterproductive work behavior [5,27]. One possible explanation may lie in differences in professional experience and work-related expectations. Younger healthcare professionals are of-ten still developing their professional careers and may face greater uncertainty and demanding work requirements. Middle-aged employees, on the other hand, may occupy more established professional positions while simultaneously carrying greater organizational and interpersonal responsibilities. These differences may contribute to variation in how reduced discretionary engagement is associated with organizational and interpersonal behavior. However, these interpretations remain tentative and should be examined more directly in future research.

### 4.1. Theoretical Implications

From a theoretical perspective, the contribution of this study is not limited to confirming that quiet quitting is related to counterproductive work behavior. The findings show that the relationships differ across specific CWB outcomes. Quiet quitting was positively associated with organizational and interpersonal CWB and with Withdrawal, whereas no statistically significant relationship was identified with Sabotage. This differentiated pattern supports the view that quiet quitting and CWB are conceptually related but not equivalent constructs. The findings also contribute to a more specific application of social exchange theory to quiet quitting. Rather than suggesting that an unfavorable employee–organization exchange produces a uniform negative behavioral response, the results are consistent with the view that employee responses may vary according to their intensity and target. Restricting discretionary effort may coexist with organizational, interpersonal, and withdrawal-related behaviors without necessarily being associated with more deliberately destructive behavior such as Sabotage. In this respect, the non-significant Sabotage relationship helps define the theoretical boundary of quiet quitting rather than merely representing an unsupported hypothesis.

The healthcare setting provides an additional contextual contribution. Healthcare work relies strongly on coordination and interdependence among professionals. When employees reduce initiative and willingness to undertake responsibilities beyond formal requirements, some work demands may be transferred to colleagues. This provides a possible contextual mechanism for the strong relationship identified between quiet quitting and CWB directed toward other employees. The healthcare context therefore does not function only as the setting from which the sample was drawn but may shape how reduced discretionary engagement is associated with interpersonal and organizational behavior.

### 4.2. Practical Implications

From a practical perspective, the findings indicate that healthcare managers and human resource professionals should not consider quiet quitting only as a productivity-related issue. Its association with organizational, interpersonal, and withdrawal-related forms of CWB suggests that reduced discretionary engagement may coexist with broader workplace problems. Managers should therefore pay particular attention to working conditions that may reduce employees’ willingness to contribute beyond formally required tasks, including excessive workload, insufficient organizational support, limited recognition, and strained interpersonal relationships. The strong relationship between quiet quitting and CWB directed toward other employees is particularly relevant in healthcare organizations, where effective service delivery depends on cooperation and coordination among professionals. Repeated situations in which employees restrict initiative or avoid additional responsibilities may place additional demands on coworkers and contribute to tensions within teams. Managerial practices aimed at improving fairness, support, communication, workload distribution, and recognition may therefore be relevant for maintaining both employee engagement and constructive interpersonal relationships.

### 4.3. Limitations

This study has several limitations. First, the cross-sectional design does not allow temporal precedence or causal relationships between quiet quitting and counterproductive work behavior to be established. Although potential endogeneity was assessed using the Gaussian copula approach, reverse relationships and the influence of unobserved common antecedents cannot be excluded. Longitudinal research would therefore be useful for examining how quiet quitting and different forms of CWB are related over time.

Second, convenience sampling was used, which limits the statistical generalizability of the findings. Respondents were recruited from ten public healthcare institutions at primary, secondary, and tertiary levels of healthcare in Central Serbia and on different weekdays, which increased the heterogeneity of the sample. Nevertheless, the findings should be generalized with caution. In addition, because refusals to participate were not systematically recorded, a response rate could not be calculated.

Third, all variables were assessed using self-reported data collected from the same respondents at one point in time. Harman’s single-factor test did not indicate that common method bias represented a major concern according to this diagnostic, but common method variance cannot be completely excluded. Future studies could combine self-reported measures with other data sources or temporally separated measurements.

Finally, the professional category was not collected because healthcare professionals were treated as the target population as a whole. Therefore, it is not possible to determine whether some of the gender differences observed in the multigroup analysis partly reflect occupational composition. Future studies could distinguish between physicians, nurses, and other healthcare professionals and examine whether the relationships identified in this study differ across professional groups.

Although the final sample included 517 participants, the number of participants in some subgroups, particularly men, was relatively limited, which may affect the stability of subgroup comparisons. In addition, although age and gender were examined in the multigroup analysis, other potentially important confounders, including professional and workplace-related characteristics, were not collected and could not be controlled.

## 5. Conclusions

This cross-sectional study found that quiet quitting was positively associated with counterproductive work behavior directed toward the organization and other employees, as well as with Withdrawal, whereas no statistically significant association was identified with Sabotage. These findings indicate that quiet quitting is related to several, but not all, forms of counterproductive work behavior and should not be considered equivalent to CWB. Differences in these relationships were also observed across gender and age groups, although these findings should be interpreted with caution because professional category was not collected and may partly account for some subgroup differences. Overall, the findings are consistent with social exchange theory, and emphasize the significance of both organizational and interpersonal outcomes in the study of quiet quitting among health care professionals. The cross-sectional design suggests the results should be interpreted as associations and not evidence of causality.

## Figures and Tables

**Table 1:** Respondents’ characteristics.

Category	Frequency	%
Gender		
Female	440	85.11
Male	77	14.89
Age		
<40	221	42.74
40–50	151	29.21
>51	145	28.05
Notes: *N* = 517		

**Table 2:** Measurement model and constructs.

Construct and Items	Loading	VIF	α	Composite Reliability	AVE
QQ: Quiet quitting			0.916	0.919	0.632
QQ01	0.742	1.998			
QQ02	0.774	1.998			
QQ03	0.851	2.810			
QQ05	0.715	1.773			
QQ06	0.823	3.388			
QQ07	0.773	2.141			
QQ08	0.848	2.802			
QQ09	0.822	3.391			
SAB: Sabotage			0.700	0.745	0.620
SAB01	0.806	1.440			
SAB02	0.852	1.377			
SAB03	0.695	1.306			
WD: Withdrawal			0.820	0.831	0.652
WD01	0.791	1.609			
WD02	0.719	1.432			
WD03	0.878	2.400			
WD04	0.832	2.119			
CWB-O: Counterproductive work behavior-Organization			0.801	0.803	0.557
CWB-O02	0.698	1.333			
CWB-O03	0.781	1.806			
CWB-O04	0.751	1.759			
CWB-O06	0.792	1.739			
CWB-O07	0.705	1.445			
CWB-P: Counterproductive work behavior-Person			0.796	0.804	0.622
CWB-P01	0.734	1.462			
CWB-P02	0.848	1.962			
CWB-P03	0.756	1.585			
CWB-P05	0.811	1.614			

**Table 3:** Discriminant validity (HTMT^0.85^ criterion).

Constructs	1	2	3	4	5
1. CWB-O: Counterproductive work behavior-Organization	–				
2. CWB-P: Counterproductive work behavior-Person	0.755				
3. QQ: Quiet quitting	0.695	0.802			
4. SAB: Sabotage	0.592	0.499	0.495		
5. WD: Withdrawal	0.837	0.791	0.754	0.675	–

**Table 4:** Coefficients of determination.

Construct	*R* ^2^
CWB-O: Counterproductive work behavior–Organization	0.369
CWB-P: Counterproductive work behavior–Person	0.484
SAB: Sabotage	0.173
WD: Withdrawal	0.438

**Table 5:** Gaussian copula assessment.

Copula Term → Outcome	β	t	*p*
GC(QQ) → CWB-O	−0.871	2.010	0.044
GC(QQ) → CWB-P	−1.075	2.421	0.016
GC(QQ) → Withdrawal	−0.808	1.915	0.056
GC(QQ) → Sabotage	−0.165	0.320	0.749

**Table 6:** Structural relationships and hypothesis testing.

Relationship	β	*t*	*p*	95% CI (Percentile Bootstrap)	Results	Hypothesis
QQ → CWB-O	1.471	3.403	<0.001	[0.433, 2.142]	Supported	H1a
QQ → CWB-P	1.760	4.003	<0.001	[0.742, 2.465]	Supported	H1b
QQ → SAB	0.580	1.130	0.259	[−0.479, 1.562]	Not supported	H1d
QQ → WD	1.463	3.468	<0.001	[0.384, 2.060]	Supported	H1c

Notes: QQ, quiet quitting; CWB-O, counterproductive work behavior-organization; CWB-P, counterproductive work behavior-person; SAB, sabotage; WD, withdrawal.

**Table 7:** Results of multigroup partial least squares path modeling.

Relationship	Path Coefficient	*p*-Value	Path Coefficient	*p*-Value	Path Coefficient	*p*-Value	Invariant
	Female	Female	Male	Male			
QQ → CWB-O	1.664	0.001 **	0.667	0.341			No
QQ → CWB-P	1.761	0.000 ***	0.915	0.068			No
QQ → SAB	0.778	0.179	0.175	0.786			N/I
QQ → WD	1.660	0.001 **	0.731	0.280			No
	Age < 40	Age < 40	Ages 41–50	Ages 41–50	Age > 51	Age > 51	
QQ → CWB-O	1.592	0.006 **	0.758	0.205	−0.848	0.242	No
QQ → CWB-P	1.370	0.011 *	1.956	0.000 ***	0.675	0.447	No
QQ → SAB	0.232	0.701	0.348	0.582	1.529	0.161	N/I
QQ → WD	1.727	0.001 **	0.428	0.457	0.961	0.239	No

Notes: QQ, quiet quitting; CWB-O, counterproductive work behavior-organization; CWB-P, counterproductive work behavior-person; SAB, sabotage; WD, withdrawal. * *p* ˂ 0.05; ** *p* ˂ 0.01; *** *p* ˂ 0.001. N/I = not interpreted because compositional invariance was not established in MICOM Step 2. The ‘Invariant’ column refers to the formal PLS-MGA/permutation comparison of structural path coefficients.

## Data Availability

The data presented in this study are available on request from the corresponding author due to ethical reasons.
